# Insights into Lead Toxicity and Detoxification Mechanisms in the Silkworm, *Bombyx mori*

**DOI:** 10.3390/insects16070699

**Published:** 2025-07-07

**Authors:** Dan-Dan Bian, Yan-Xia Shi, Kai-Wen Shi, Hui-Cong Du, Bo-Ping Tang, Qiu-Ning Liu

**Affiliations:** 1 Jiangsu Key Laboratory for Bioresources of Saline Soils, Jiangsu Synthetic Innovation Center for Coastal Bio-Agriculture, Jiangsu Provincial Key Laboratory of Coastal Wetland Bioresources and Environmental Protection, School of Wetlands, Yancheng Teachers University, Yancheng 224007, China; 2 Anhui Key Laboratory of Resource Insect Biology and Innovative Utilization, College of Life Sciences, Anhui Agricultural University, Hefei 230036, China; 3 College of Biotechnology and Pharmaceutical Engineering, Nanjing University of Technology, Nanjing 210009, China

**Keywords:** lead, *Bombyx mori*, detoxication, oxidative stress, transcriptome, CncC, RNAi

## Abstract

*Bombyx mori*, a key lepidopteran model with economic importance, is highly susceptible to environmental heavy metal pollution. This study investigated the mechanisms of Pb toxicity and the associated detoxification and metabolic defense responses in silkworms, employing transcriptome sequencing, enzyme activity assays, and histopathological analysis. Pb exposure caused significant histopathological changes and apoptosis in the fat body, marked by structural disorganization, swollen adipocytes, and degraded extracellular matrix. Molecular analysis showed activation of antioxidant defenses, with superoxide dismutase (SOD) and catalase (CAT) activities significantly elevated (*p* < 0.05), while peroxidase (POD) activity declined (*p* < 0.05). Levels of malondialdehyde (MDA) and glutathione (GSH) also decreased. In detoxification responses, carboxylesterase (CarE) activity was reduced, whereas cytochrome P450 (P450) and glutathione S-transferase (GST) activities increased (*p* < 0.05). Transcriptome sequencing revealed 1,418 differentially expressed genes (DEGs), with notable upregulation of key detoxification genes (*p* < 0.05), including six cytochrome P450s (CYPs), five uridine diphosphate-glycosyltransferases (UGTs), three glutathione S-transferases (GSTs), and six ATP-binding cassette transporters (ABCs). KEGG enrichment analysis highlighted the involvement of these DEGs in drug metabolism, glutathione metabolism, and ABC transporter pathways (*p* < 0.05). Functional validation showed that knocking down Cap ‘n’ Collar C (CncC) significantly suppressed key detoxification genes (*CYP18A1*, *CYP332A1*, *GSTd3*, *GSTt1*, *UGT33D8*; *p* < 0.05). qRT-PCR and Western blot analyses confirmed that the Caspase-3 pathway mediates Pb-induced apoptosis, with increased cleaved Caspase-3 and Caspase-4 levels following CncC silencing. Overall, our findings elucidate the mechanisms of Pb toxicity in silkworms and identify *CncC* as a critical regulator of detoxification and defense against heavy metal stress in lepidopteran insects.

## Simple Summary

The silkworm (*Bombyx mori*), a vital species for silk production, is highly susceptible to environmental toxins such as lead (Pb). Pb contamination, entering silkworms through mulberry leaves, causes oxidative stress and triggers apoptosis in their fat body tissue. Transcriptome analysis revealed that Pb exposure significantly alters gene expression in the fat body, activating antioxidant defenses and key detoxification enzymes. We identified the transcription factor *CncC* as a central regulator of the detoxification response. Silencing the *CncC* gene reduced detoxification enzyme activity and exacerbated Pb-induced fat body damage and apoptosis. This study provides the first evidence of the CncC pathway’s critical role in mediating Pb detoxification in a lepidopteran insect, revealing key molecular mechanisms of Pb toxicity in silkworms and identifying *CncC* as a potential target for mitigating heavy metal stress in sericulture.

## 1. Introduction

The silkworm (*Bombyx mori*), a member of the Lepidoptera order and Bombycidae family, is native to China and holds significant cultural and economic importance [1,2]. It has been central to silk production for thousands of years and continues to play a vital role in the textile industry [3,4]. Beyond silk, silkworm pupae are a high-quality source of dietary protein [5,6]. However, silkworm health is vulnerable to various parasites, pathogens, and environmental factors such as heavy metal contamination [7,8,9]. Due to prolonged artificial domestication, silkworms exhibit heightened sensitivity to heavy metals, making them an ideal model for environmental toxicology research [7,10,11].

Heavy metals are ubiquitous in soil, rock, air, water, and living organisms, serving as essential micronutrients for biological growth and development [7,12,13,14,15,16]. However, they also pose significant environmental and health risks, contributing to global pollution [17]. Lead (Pb), in particular, is a highly mobile environmental pollutant that accumulates in soil and is challenging to remediate [14,18,19]. It can have severe phytotoxic effects and impact herbivorous insects through food chain transfer or direct ingestion [18,20]. Pb ions can enter the silkworm body via mulberry leaves, impairing silkworm health and causing mass mortality at high concentrations [21].

Excessive heavy metals can induce the generation of free radicals and reactive oxygen species (ROS) within cells, disrupting cytoplasmic homeostasis and leading to oxidative stress and apoptosis [17,22,23]. Insects have evolved antioxidant defense systems to counteract oxidative damage, with enzymes such as superoxide dismutase (SOD), catalase (CAT), peroxidase (POD), and glutathione S-transferases (GSTs) playing key roles in ROS scavenging [24,25,26]. Additionally, metal detoxification enzymes like cytochrome P450s (P450s), carboxylesterase (CarE), UDP-glycosyltransferase (UGT), and glutathione S-transferase (GST) can metabolize ROS into non-toxic by-products, thereby alleviating heavy metal toxicity [7,27]. The Cap ‘n’ Collar isoform-C (CncC) regulates redox balance, cell proliferation, and detoxification [4,24,28]. The CncC/Keap1 signaling pathway modulates the expression of detoxification, antioxidant, and metabolic enzymes, enhancing resistance to external stress [24,28,29]. For instance, knockdown of *Drosophila melanogaster* CncC expression significantly downregulates the detoxification-related genes *CYP6a8* and *CYP6a2* [30]. In *Tribolium castaneum*, the CncC-Maf complex enhances the transcription of P450 family genes *CYP6BQ6*, *CYP6BQ7*, and *CYP6BQ9* [31]. CncC can also regulate downstream gene expression in response to heavy metals, H_2_O_2_, and mitochondrial ROS [24,26,29].

As the primary metabolic and detoxification organ in insects, functionally analogous to the vertebrate liver, the fat body exhibits heightened sensitivity to environmental stressors, including heavy metals [7,15,32]. To investigate the molecular mechanisms underlying Pb-induced toxicity in *B. mori*, we conducted a comprehensive transcriptome analysis of the fat body. By comparing silkworms fed normal mulberry leaves with those exposed to Pb, we identified key differentially expressed genes (DEGs) that regulate physiological adaptation and detoxification responses. These findings provide valuable insights into mitigating lead toxicity in sericulture and offer a basis for ecological risk assessments of Pb contamination in agricultural ecosystems.

## 2. Materials and Methods

### 2.1. Insect Rearing and Sample Preparation

Silkworm larvae (strain ‘Jingsong’) were reared at 25–27 °C and 75–85% humidity under a 12 h light-dark cycle. From the third day of the fifth instar, experimental groups were fed mulberry leaves soaked in a 0.4 g/L Pb solution (prepared from analytical grade Pb(NO_3_)_2_), while control groups received leaves soaked in ddH_2_O [33]. The leaves were fed to the larvae after brief air-drying. Each treatment included three replicates of 100 larvae. After 48 h, larvae were dissected in ice-cold physiological saline (0.75% NaCl, pH 7.4). Fat bodies were collected, rinsed in saline, and stored at −80 °C. The Pb concentration was chosen based on our prior study [21], which demonstrated significant physiological effects in silkworms at this concentration.

### 2.2. Histological and TUNEL Assay Analysis of Pb-Exposed Fat Bodies

Fat body tissues were collected from control and Pb-treated groups after 48 h of exposure for histopathological evaluation. Tissues were fixed in 4% neutral buffered formalin overnight at 4 °C. After fixation, tissues were washed in PBS (3 × 15 min), dehydrated through a graded ethanol series, cleared in xylene, and embedded in paraffin wax. Serial 5 μm thick longitudinal sections were cut and mounted on glass slides. Sections were deparaffinized, rehydrated, and stained with hematoxylin–eosin (H&E). Histopathological evaluation was performed under an optical microscope (Nikon U-III Multi-point Sensor System) to document alterations such as vacuolization, nuclear condensation/fragmentation, and cell density changes.

Apoptotic cells were detected in adjacent sections using the In Situ Cell Death Detection Kit, Fluorescein. Sections were treated with 20 μg/mL DNase-free proteinase K at 37 °C for 20 min. Sections were then incubated with TUNEL reaction mixture in a humidified chamber at 37 °C in the dark for 60 min. Negative controls were incubated with label solution only, while positive controls were treated with DNase I prior to the TUNEL reaction. After washing, sections were counterstained with DAPI and mounted with anti-fade fluorescent mounting medium. Images were acquired using a fluorescence microscope (IX71, Olympus, Tokyo, Japan) equipped with FITC and DAPI filter sets. TUNEL-positive cell density was quantified by counting green-fluorescent cells per mm^2^ in five random fields per slide at 400× magnification.

### 2.3. Enzyme Activity Determination

At 48 h post-exposure initiation, fat body tissues were dissected from silkworms in both the control and Pb-exposed groups (n = 3). Approximately 100 mg of tissue (wet weight) per sample was immediately homogenized on ice in 900 μL of ice-cold 0.9% (*w*/*v*) NaCl solution using an ultrasonic cell disruptor (200 W, pulsed mode: on 2 s/off 3 s, total duration: 30 s). The homogenate was centrifuged at 10,000× *g* for 15 min at 4 °C, and the resulting supernatant was collected, aliquoted, and stored at −80 °C.

Protein concentration in the supernatant was determined using the Bradford assay kit (Nanjing Jiancheng Institute of Bioengineering, Nanjing, China, Cat. No. A045-2-2) with bovine serum albumin as the standard. Enzyme activities (superoxide dismutase (Cat. No. A001-3-2), catalase (Cat. No. A007-1-1), peroxidase (Cat. No. A084-3-1), glutathione S-transferase (Cat. No. A004-1-1), carboxylesterase (Cat. No. A133-1-1), caspase-3 (Cat. No. H076), caspase-4 (Cat. No. H077)) and biochemical levels (malondialdehyde (Cat. No. A003-1-2), reduced glutathione (Cat. No. A006-2-1)) were assayed using kits from Nanjing Jiancheng Institute of Bioengineering. Cytochrome P450 content was determined with an ELISA kit (Cat. No. BY-PC20457; Baiyi Biotechnology Co., Ltd. (Hangzhou, China)). All assays followed the manufacturers’ protocols, using colorimetric, fluorometric, or ELISA methods, and were measured with a microplate reader (Tecan Infinite M200 PRO (Shanghai, China)). Each sample was analyzed in triplicate. Enzyme activities are expressed as U/mg prot, malondialdehyde as mmol/mg prot, reduced glutathione as mmol/mg prot, and cytochrome P450 as nmol/min/mg prot, calculated based on kit-provided standard curves.

### 2.4. Total RNA Isolation, cDNA Library Construction, and Sequencing

Fat body tissues were collected from control and Pb-exposed silkworms 48 h post-exposure. For each of the three biological replicates per group, tissues from five larvae were pooled. Total RNA was extracted using TRIzol™ reagent (Invitrogen, Carlsbad, CA, USA) as per the manufacturer’s instructions. RNA quality was verified via 1% agarose gel electrophoresis, and its purity, concentration, and integrity were quantified using a NanoDrop spectrophotometer (Thermo Fisher Scientific, Waltham, MA, USA; OD260/280 ratios: 1.8–2.2; OD260/230 ratios > 1.8), Qubit 2.0 Fluorometer (Life Technologies, Carlsbad, CA, USA), and Agilent 2100 Bioanalyzer (Agilent Technologies, Santa Clara, CA, USA), respectively. Only RNA samples with RIN ≥ 7.0 were used for subsequent analysis.

cDNA libraries were constructed from 1 µg of total RNA using the TruSeq Stranded mRNA Library Prep Kit (Illumina, San Diego, CA, USA). mRNA was enriched with Oligo(dT) magnetic beads, fragmented, and reverse-transcribed into double-stranded cDNA. After end-repair, 3′-end adenylation, and ligation of Illumina sequencing adapters, the cDNA was amplified by PCR and purified with AMPure XP beads (Beckman Coulter, Brea, CA, USA). Library size distribution and concentration were validated using an Agilent 2100 Bioanalyzer. To minimize batch effects, all libraries were prepared in a single batch and randomized across sequencing lanes. Sequencing was performed on an Illumina NovaSeq 6000 platform (SP or SX flow cell) at Beijing Biomarker Technologies Co., Ltd. (Beijing, China), generating 150 bp paired-end reads with a target depth of 40 million reads per sample.

### 2.5. De novo Assembly and Functional Annotation

Trimmomatic (v0.39) was used to remove low-quality reads (average quality score < 20) and adapter-containing reads (adapter contamination > 2%), yielding high-quality clean reads. FastQC (v0.11.9) was employed to assess the Q30 score, G+C content, and sequence repeat levels. Trinity (v2.8.4) was used for de novo assembly with a k-mer length of 25, -min_kmer_cov 2, and -normalize_reads enabled. The assembly quality was evaluated by contig N50 and scaffold N50. TransDecoder (v5.7.0) predicted the coding regions and amino acid sequences of unigenes. BLAST (v2.10.1) was used to align unigenes against multiple databases (Nr, Swiss-Prot, Pfam, eggNOG, GO, and KEGG) with an E-value threshold of 1e-5 for functional annotation.

### 2.6. Differentially Expressed Genes and Gene Enrichment Analysis

Bowtie [34] was employed to align the sequencing reads from each sample against the unigene library. Based on these alignment results and using RSEM [33], the gene expression levels for each sample were estimated. Gene expression levels were quantified using the FPKM (fragments per kilobase per million) value [11], which enabled direct comparison of expression differences between samples. DEGSeq was used to analyze differential expression between sample groups and identify DEGs. Genes with FDR < 0.05 and |Fold Change| ≥ 2 were deemed differentially expressed. Subsequently, GO functional enrichment and KEGG pathway enrichment analyses of DEGs were conducted using GOSeq R [33] and KOBAS [35], respectively, to further explore the potential roles of DEGs.

### 2.7. Expression Validation of DEGs from RNA-Seq by qRT-PCR

DEGs were identified from the 48 h control (CK) versus the 48 h lead (Pb)-exposed group. Primers for these DEGs, designed using Primer Premier 5.0 software (Premier Biosoft, Palo Alto, CA, USA), are listed in Table S1. Total RNA was extracted from each sample (with three biological replicates) using the RNAiso Plus kit (Takara, Tokyo, Japan, Cat. No. 9108) following the manufacturer’s instructions, then reverse-transcribed into cDNA using the PrimeScript^®^ RT reagent Kit with gDNA Eraser (Takara, Tokyo, Japan, Cat. No. RR047A). The synthesized cDNA served as the template for qRT-PCR. The Ct value of the target gene was measured using the ChamQ Universal SYBR qPCR Master Mix (Vazyme, Cat. No. Q711) kit on the 7500 Fast Real-Time PCR System (Applied Biosystems, Foster City, CA, USA, Cat. No. 7500). Actin3, which exhibited stable expression in preliminary experiments, was selected as the internal reference gene [36]. Each gene was analyzed in triplicate, and relative expression levels were calculated using the 2^−ΔΔCt^ method [4].

### 2.8. Western Blotting Analysis

The fat body (100 mg) was homogenized in a mixture of IP cracking solution and PMSF (100 mM, 100:1 ratio) on ice. After centrifugation at 4 °C and 12,000 rpm for 15 min, the supernatant was collected, and protein concentration was measured using the BCA method (Beyotime, Shanghai, China). Forty micrograms of protein were separated by 12.5% SDS-PAGE and transferred to a PVDF membrane (Merck Millipore, Darmstadt, Germany). The membrane was blocked with 5% non-fat milk in TBST at room temperature for 1 h, then incubated with primary antibodies overnight at 4 °C. After washing, the membrane was incubated with the secondary antibody (HRP-labeled goat anti-immune IgG, Beyotime, Shanghai, China) at room temperature for 1 h. Signals were visualized using a developer drop (Beyotime, Shanghai, China), and bands were quantified using ImageJ software v1.x (NIH, Bethesda, MD, USA,). The primary antibodies used were β-tubulin (TransGen Biotech, Beijing, China), Caspase-3 (GenScript, Nanjing, China), Caspase-4 (GenScript, Nanjing, China), Cytc (HuaAn Biological, Hangzhou, China), and Bax (Servicebio, Wuhan, China).

### 2.9. dsRNA-Mediated Gene Silencing

Fragments of the CncC gene and GFP were amplified by PCR using primers (Table S1) with the T7 promoter sequence (5′-TAATACGACTCACTATAGG-3′) at the 5′ ends. The PCR products were purified using a Gel Extraction/PCR Purification Kit (Qiagen, Hilden, Germany) and used as templates for in vitro transcription. Double-stranded RNA (dsRNA) templates for CncC (dsCncC) and GFP (dsGFP) were synthesized using the T7 RNAi Transcription Kit (Vazyme, Nanjing, China). The dsRNA quality and size were confirmed by 1% agarose gel electrophoresis, showing single, sharp bands. The concentration and purity of dsRNA were determined using a NanoDrop 2000 spectrophotometer (Thermo Fisher Scientific, USA), with A260/A280 ratios between 1.8 and 2.0. Based on preliminary experiments, the dsRNA stocks were diluted to an optimal working concentration of 1 μg/μL with nuclease-free water to ensure efficient gene silencing [37].

Fifth-instar silkworm larvae on day 3, with uniform size and developmental stage, were selected for injection. The larvae were anesthetized on ice for 3–5 min, and the injection site was disinfected with 70% ethanol. Approximately 10 μg of dsRNA (10 μL) was injected into the hemolymph using a glass capillary needle attached to a microinjector (Drummond Scientific, Broomall, PA, USA). The dsGFP-injected group served as a control, with three independent biological replicates for each group [38]. Post-injection, the larvae were maintained at 25 ± 1 °C, 75% humidity, and a 12 h light/dark cycle with fresh mulberry leaves. At 24 and 48 h post-injection, 15 silkworms per group were sampled to assess gene silencing efficiency and measure the expression of detoxification-related genes (e.g., *CYP12A2*, *CYP332A1*) using RT-qPCR. Enzyme activities were also measured using standard biochemical assays.

### 2.10. Statistical Analysis

Statistical analysis was performed using IBM SPSS Statistics 24.0 (IBM, Armonk, NY, USA). All experimental data are presented as mean ± standard error of the mean (SEM) with n = 3. To compare differences between the experimental and control groups, an independent samples *t*-test was employed. GraphPad Prism 8 (San Diego, CA, USA) was used for graphical representation. Significance levels were defined as *p* < 0.05 (*), *p* < 0.01 (**), and *p* < 0.001 (***), indicating significant differences between groups, whereas *p* ≥ 0.05 denoted no significant difference (ns).

## 3. Results

### 3.1. Pb Exposure Results in Fat Body Tissue Damage and Cellular Apoptosis in B. mori

**Figure 1:**
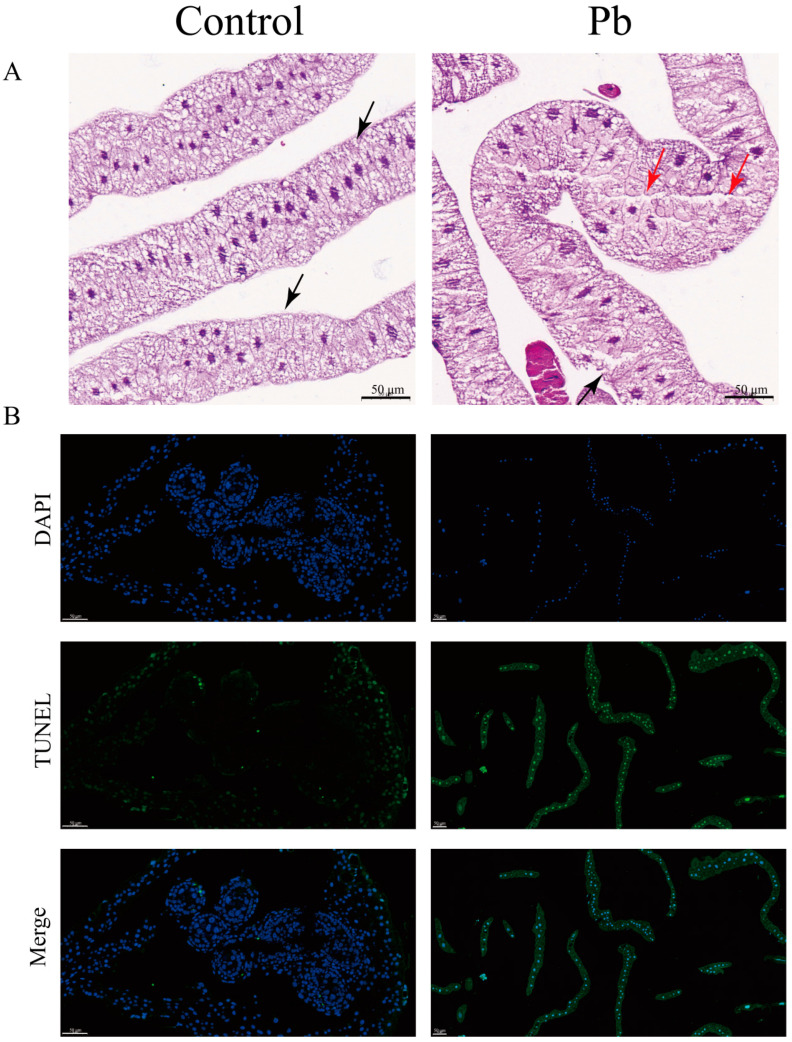
Histopathological alterations and apoptosis induction in *B. mori* fat body following Pb exposure. (**A**) Representative hematoxylin and eosin (H&E) stained sections of fat body tissue from control (left) and Pb-exposed (right) silkworms after 48 h. Red and black triangles indicat some vacuoles and film of fat cells (bar = 50 μm). (**B**) Apoptotic cells in the fat body were detected using the TUNEL assay (green fluorescence) with nuclei counterstained with DAPI (blue fluorescence). Merged images show overlapping signals. In the control group, few TUNEL-positive cells are observed. However, Pb-exposed groups show a significant increase in TUNEL-positive cells, indicating elevated apoptosis (bar = 50 μm).

Histopathological analysis of the fat body from *B*. *mori* larvae exposed to Pb for 48 h revealed significant architectural and cellular alterations. In the control group, the fat body exhibited a well-organized structure with distinct cell nuclei, a clear basal lamina boundary, and densely packed cells arranged in an orderly manner. However, in Pb-exposed larvae, the fat body displayed marked disorganization. Adipocytes appeared swollen and exhibited apoptotic morphology, characterized by a blurred basal lamina and compromised cell integrity. The extracellular matrix also showed signs of degradation, appearing highly porous and vacuolated (Figure 1A).

In addition, to gain deeper insights into the cytological alterations caused by Pb exposure, we carried out TUNEL assay experiments. The results further confirmed a remarkable increase in apoptotic cells within the Pb-treated fat body. These cells displayed typical apoptotic features, such as shrinkage and chromatin condensation. Collectively, these findings provide evidence that Pb exposure induces severe tissue damage and triggers cellular apoptosis in the silkworm fat body *(*Figure 1B).

### 3.2. Molecular Insights into Pb-Induced Apoptosis in the Silkworm Fat Body

**Figure 2:**
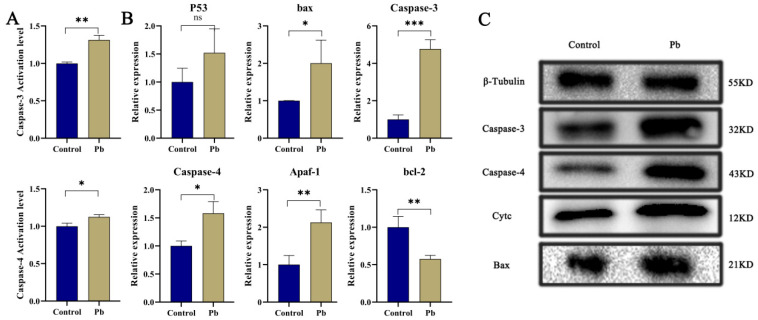
Pb-Induced Apoptosis in the Fat Body of *B. mori*. (**A**) Relative enzymatic activities of Caspase-3 and Caspase-4 in fat body tissue of B. mori after 48 h of Pb exposure. (**B**) Relative mRNA expression levels of apoptosis-related genes after 48 h of Pb exposure. Pro-apoptotic genes (*p53*, bax, *Caspase-3*, *Caspase-4*, *Apaf-1*) showed significant upregulation, while the anti-apoptotic gene bcl-2 exhibited significant downregulation. (**C**) Western blot analysis of apoptosis-related proteins. Pb exposure significantly increased the levels of cleaved Caspase-3, Caspase-4, Cytochrome c, and Bax. β-Tubulin served as the loading control to ensure equal protein loading. Data are presented as mean ± SD. (* *p* < 0.05, ** *p* < 0.01, *** *p* < 0.001, n = 3).

Apoptosis, a meticulously regulated mechanism of programmed cell death, plays a pivotal role in preserving cellular homeostasis [6,7,26]. Our findings reveal that exposure to Pb markedly elevates the activities and protein levels of Caspase-3 and Caspase-4 within the silkworm fat body (Figure 2A,C), signaling significant cellular stress and the activation of apoptosis. When contrasted with the control group, the Pb-exposed group exhibits substantially higher activities of Caspase-3 and Caspase-4, alongside a conspicuous upregulation of these caspases at the protein level. This evidence strongly implies that Pb instigates the activation of key caspases, thereby precipitating apoptosis. Further investigation shows that Pb exposure leads to the upregulation of pro-apoptotic genes, including *p53*, *Bax*, *Caspase-3*, *Caspase-4*, and *Apaf-1*, while concurrently downregulating the anti-apoptotic gene *bcl-2* (Figure 2B), a shift that unequivocally steers the cellular process toward apoptosis.

In the Pb-exposed group, heightened protein levels of Cytc, Caspase-3, Caspase-4, and Bax are also observed (Figure 2C). The rise in Cytc signifies increased mitochondrial membrane permeability [39], which is likely to set off the formation of apoptosomes and the subsequent activation of the Caspase cascade [40,41]. The upregulation of Bax further corroborates the activation of the mitochondrial pathway, given that Bax facilitates the permeabilization of the mitochondrial outer membrane. Overall, these results collectively furnish molecular proof that Pb induces apoptosis in the silkworm fat body by modulating *Bax* and *bcl-2* to activate the mitochondrial pathway and upregulating *p53* to trigger DNA damage responses.

### 3.3. Pb Exposure Triggers Oxidative Stress and Activates Detoxification Enzymes

**Figure 3:**
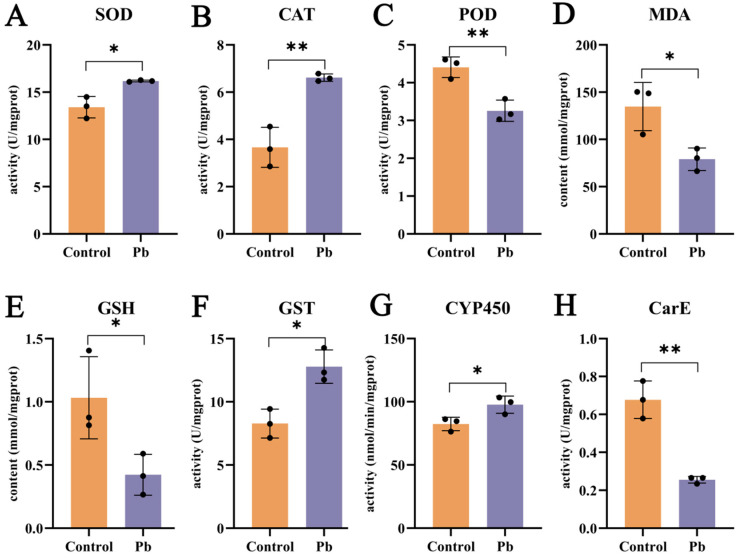
Effects of lead exposure on fat body biochemistry in B. mori. (**A**–**E**) Oxidative stress markers: Activities of superoxide dismutase (SOD), catalase (CAT), and peroxidase (POD), and contents of malondialdehyde (MDA) and glutathione (GSH). (**F**–**H**) Detoxification enzyme activities: Glutathione S-transferase (GST), cytochrome P450 (CYP450), and carboxylesterase (CarE). Data are expressed as mean ± SEM (n = 3). Asterisks indicate significant differences compared to the control group (* *p* < 0.05, ** *p* < 0.01).

Pb exposure exerts a substantial influence on the oxidative stress and detoxification enzyme systems in the silkworm fat body. After 48 h of Pb treatment, the activities of superoxide dismutase (SOD) and catalase (CAT) are elevated, whereas peroxidase (POD) activity is reduced (Figure 3A–C). This indicates that the antioxidant defense system is activated in response to Pb-induced oxidative stress. The increase in SOD activity, which catalyzes the dismutation of superoxide to hydrogen peroxide (H_2_O_2_), is expected to lead to higher H_2_O_2_ levels [26,33,42,43]. Consequently, the upregulation of CAT activity becomes necessary to break down the accumulated H_2_O_2_. The observed decrease in malondialdehyde (MDA) content supports the conclusion that oxidative stress has been alleviated (Figure 3D). However, the reduction in glutathione (GSH) levels implies that GSH may be consumed in the formation of GSH–metal complexes upon Pb exposure (Figure 3E).

In terms of detoxification, carboxylesterase (CarE) activity decreases at 48 h (Figure 3H), suggesting its primary role in the early phase of detoxification. By contrast, the activities of cytochrome P450 monooxygenases (P450) and glutathione S-transferase (GST) are enhanced (Figure 3F,G). P450 enzymes are mainly involved in Phase I of xenobiotic metabolism, while GST enzymes play a key role in Phase II [27,31,44]. The increased activities of these two enzyme families highlight their importance in the detoxification process. In summary, Pb exposure induces oxidative stress and activates detoxification enzymes in the silkworm fat body, with the increased activities of P450 and GST being particularly significant in the detoxification response.

### 3.4. Transcriptome Sequencing, Assembly, and Annotation

High-quality transcriptome sequencing of the silkworm fat body was conducted to provide a robust foundation for downstream analyses. For Pb-exposed samples, 21,267,028 clean reads (6.37 Gb) were obtained, compared to 21,816,830 clean reads (6.53 Gb) for control samples. The sequencing quality was excellent, with Q30 values exceeding 95% and a comparable GC content of approximately 47.6% in both groups (Table S2). Alignment against the reference genome achieved rates over 92% for both groups, with uniquely mapped reads accounting for 88.45–89.72% (Table S3). These results confirm the high quality and reliability of the data for subsequent differential expression analysis.

**Figure 4:**
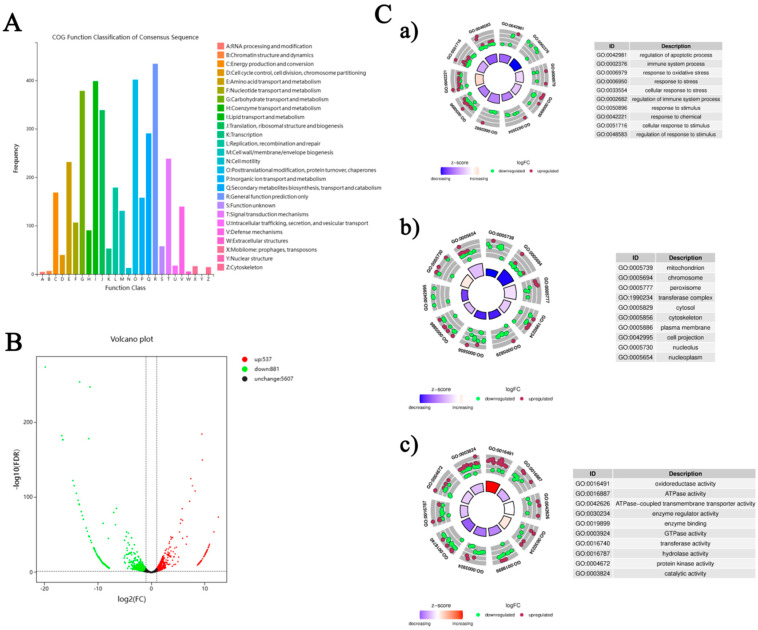
Bioinformatics analysis of the fat body transcriptome in *B. mori* following Pb exposure. (**A**) COG functional classification of assembled transcripts. The X-axis shows the 26 COG functional categories, and the Y-axis indicates the number of genes in each category. (**B**) Volcano plot of DEGs between the lead-exposed group and control group. Red dots indicate significantly up-regulated DEGs (*p* < 0.05 and |log^2^FC| ≥ 2), green dots indicate significantly down-regulated DEGs, and black dots represent non-significantly expressed genes. (**C**) Gene Ontology (GO) enrichment analysis of DEGs: (**a**) Biological Process, (**b**) Cellular Component, (**c**) Molecular Function. Bubble charts display enriched GO terms; the color scale represents the average log^2^FC values, and bubble size corresponds to the number of genes. Selected enriched GO terms with their corresponding IDs are listed.

Functional annotation of 15,842 distinct unigenes from the silkworm fat body was performed across eight databases: COG, GO, KEGG, KOG, Pfam, Swiss-Prot, eggNOG, and Nr (Table S4), providing a comprehensive functional context. Through Blast2GO, 10,692 unigenes were classified into Biological Process (BP), Cellular Component (CC), and Molecular Function (MF) categories, covering 36 subclasses. Of particular note were the 921 unigenes associated with ‘response to stimulus’ and 81 with ‘immune system process’ (BP), alongside 45 and 44 unigenes linked to ‘detoxification’ and ‘antioxidant activity’ (MF), respectively (Figure S1, Table S5). These findings highlight the silkworm fat body’s inherent stress response capabilities. COG analysis of 3,388 unigenes identified 26 functional groups, with 140 unigenes classified under ‘Defense mechanisms (V)’ (Figure 4A, Table S6), further supporting the tissue’s potential role in detoxification and antioxidant defense.

Figure 4C presents GO enrichment analysis results, showing gene regulation patterns under Pb exposure. Circular plots in subfigures a, b, and c display gene enrichment in BP, CC, and MF categories. In BP (Figure 4C-a), enrichment in terms like ‘regulation of apoptotic process’ and ‘immune system process’ implies activated cell survival and immune pathways. The CC analysis (Figure 4C-b) reveals significant enrichment in cellular compartments such as ‘mitochondrion’ and ‘cytosol’, suggesting these are key sites for Pb responses. For MF (Figure 4C-c), enriched terms like ‘oxidoreductase activity’ and ‘ATPase activity’ indicate possible changes in metabolic and energy processes. Overall, these findings provide a detailed view of the molecular mechanisms behind the silkworm fat body’s response to Pb exposure, emphasizing its role in stress adaptation and detoxification.

### 3.5. Identification and Functional Enrichment of DEGs

**Figure 5:**
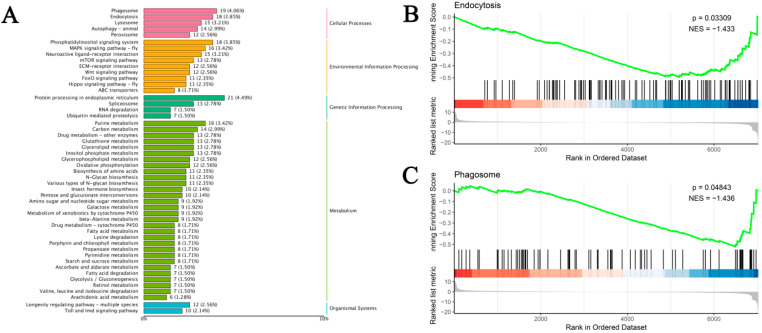
KEGG enrichment and GSEA of DEGs in *B. mori* fat body after Pb exposure. (**A**) Top enriched KEGG pathways (*p* < 0.05). Bar length represents the number of DEGs per pathway, and color indicates the KEGG pathway category. (**B**,**C**) Gene Set Enrichment Analysis (GSEA) for (**B**) endocytosis and (**C**) phagosome pathways. The green line indicates the running enrichment score, vertical black lines show gene set member positions, and the top right corner displays the normalized enrichment score (NES) and significance.

Based on the transcriptome sequencing and functional annotation of the silkworm fat body, we conducted a differential expression analysis (DEG-seq) with a threshold of FDR < 0.05 and |log2(FC)| ≥ 2. This analysis identified a total of 1418 unigenes that respond to Pb exposure, comprising 537 upregulated and 881 downregulated DEGs (Figure 4B). KEGG pathway analysis classified 1052 DEGs into 158 pathways. The top enriched pathways were closely linked to Pb toxicity and defense mechanisms. These included immune and antioxidant defense pathways such as ‘Phagosome’, ‘Endocytosis’, ‘Lysosome’, and ‘Glutathione metabolism’. Signal transduction pathways like ‘MAPK signaling pathway’ and ‘Neuroactive ligand–receptor interaction’ were also enriched. Additionally, metabolic reprogramming pathways including ‘Protein processing in endoplasmic reticulum’, ‘Purine metabolism’, and ‘Carbon metabolism’ were identified. Other enriched pathways were involved in detoxification and xenobiotic responses, such as ‘Drug metabolism-other enzymes’, ‘Metabolism of xenobiotics by cytochrome P450’, and ‘ABC transporters’ (Figure 5A).

GSEA analysis further confirmed the enrichment of ‘Endocytosis’ and ‘Phagosome’ pathways. The running enrichment score curves for these pathways showed a decreasing trend followed by an upward trend (Figure 5B). In summary, Pb exposure significantly impacts silkworm physiology by inducing extensive differential gene expression. DEGs are highly enriched in pathways related to immune responses, antioxidant defense, detoxification, signal transduction, and essential metabolic processes, reflecting a complex cellular response. The activation of specific detoxification genes may explain how silkworms counteract Pb toxicity, while metabolic alterations could account for the observed adverse effects on growth and development. These findings enhance our understanding of the molecular mechanisms underlying silkworms’ response to Pb stress and provide a foundation for future research aimed at mitigating heavy metal stress in silkworms.

### 3.6. Heatmap Analysis and Quantitative Validation

**Figure 6:**
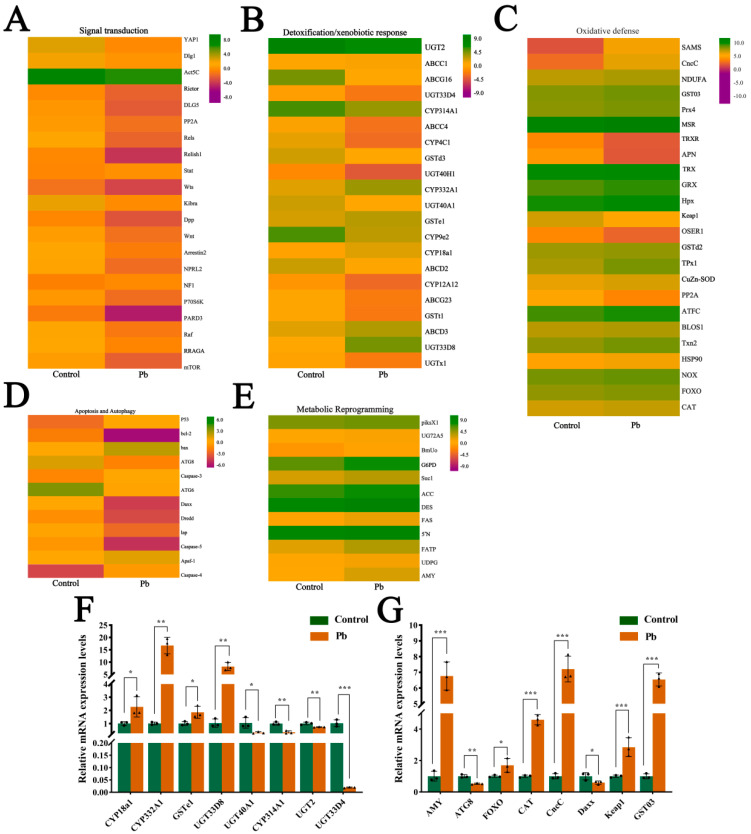
Expression profiling and validation of lead-responsive genes in *B. mori* fat body. (**A**–**E**) Heatmaps of DEGs categorized by functional groups: (**A**) Signal transduction, (**B**) Detoxification/xenobiotic metabolism, (**C**) Oxidative stress defense, (**D**) Apoptosis/autophagy, (**E**) Metabolic reprogramming. Color scale: red indicates up-regulation, green indicates down-regulation compared to the control. Rows represent individual genes clustered by functional annotation. (**F**,**G**) qPCR validation of representative DEGs. mRNA levels in the control versus Pb-exposed groups. Data are presented as mean ± SEM (n = 3 biological replicates); * *p* < 0.05, ** *p* < 0.01, *** *p* < 0.001 (Student’s *t*-test).

We performed KEGG enrichment and UniProt annotation, and visualized DEG expression patterns in key pathways via heatmaps. These pathways included antioxidant defense (e.g., glutathione metabolism, peroxisome, ubiquitin-mediated proteolysis), signal transduction (e.g., MAPK, mTOR, Wnt, Foxo, Hippo pathways), metabolic reprogramming (e.g., carbon, purine, amino sugar/nucleotide sugar, starch/sucrose, galactose, lipid metabolism pathways), and detoxification/xenobiotic response (e.g., drug metabolism-other enzymes, cytochrome P450 metabolism, ABC transporters, lysosome pathways) (Figure 6A–E). Results showed that Pb exposure induced widespread transcriptional reprogramming, with key defense and adaptation genes being significantly activated or repressed. Specifically, we identified 21 DEGs in signal transduction pathways (Figure 6A), 21 in detoxification/xenobiotic response pathways (Figure 6B), 24 in oxidative defense pathways (Figure 6C), 12 in apoptosis and autophagy pathways (Figure 6D), and 12 in metabolic reprogramming pathways (Figure 6E).

In the signal transduction pathways, genes such as *YAP1* were upregulated, while *Rictor* and *Relish1* were downregulated. In the detoxification/xenobiotic response pathways, most genes were upregulated, including *UGT2* and *ABCC1*. Similarly, in the oxidative defense pathways, the majority of genes were upregulated, such as *SOD* and *TRX*. However, in the apoptosis and autophagy pathways, the gene expression changes were more complex, with some genes such as *p53* and *Bax* being upregulated, while others, such as *ATG8* and *ATG6,* were downregulated. In the metabolic reprogramming pathways, there were both upregulated genes (e.g., *piksX1*, *UG72A5*) and downregulated genes (e.g., BmUo). To validate the accuracy of the transcriptome results, we randomly selected 16 genes for qRT-PCR validation (Figure 6F,G). The results confirmed the RNA-seq findings, thereby verifying the accuracy of the high-throughput sequencing data. Overall, the varying gene expression changes in different pathways reflect the complex regulatory patterns of multiple physiological processes in the silkworm fat body under Pb exposure.

### 3.7. CncC Regulates Detoxification Gene Expression in Response to Pb Stress

**Figure 7:**
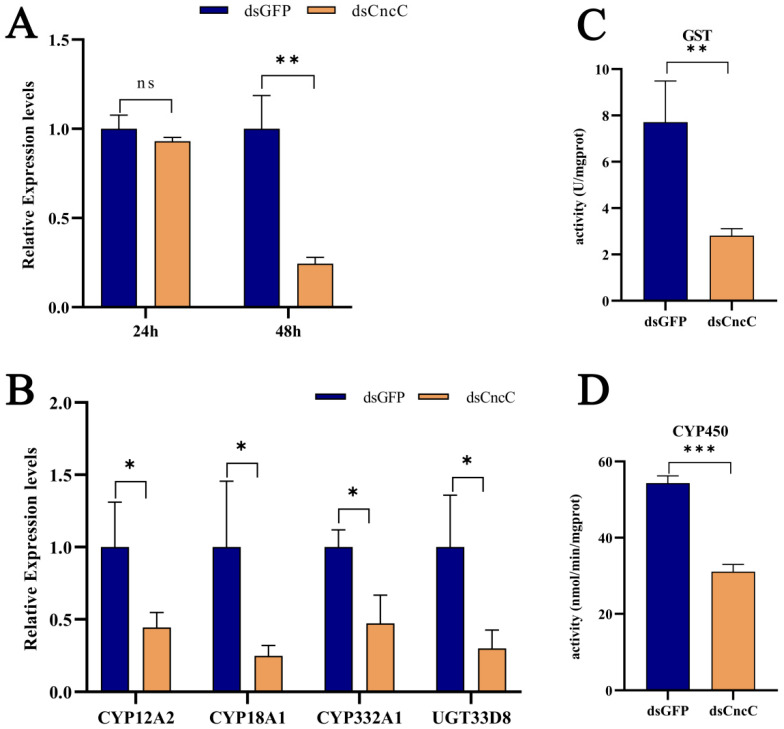
Regulatory role of CncC in lead-responsive detoxification genes in *B. mori* fat body after RNAi knockdown. (**A**) Temporal expression of CncC in dsCncC versus dsGFP control groups at 24 h and 48 h post-treatment. (**B**) Expression profiles of individual detoxification genes in dsCncC versus dsGFP controls. (**C**) GST enzyme activity in dsCncC and dsGFP groups. (**D**) CYP450 enzyme activity in dsCncC and dsGFP groups. Data are presented as mean ± SEM (n = 3 biological replicates). Asterisks indicate significant differences compared to the dsGFP control (* *p* < 0.05, ** *p* < 0.01, *** *p* < 0.001; Student’s *t*-test).

CncC, a key transcription factor, plays a central role in insect detoxification [26,28,45,46]. Our transcriptome analysis revealed that Pb exposure significantly upregulated *CncC* (with a 7.21-fold increase, *p* < 0.001) and its negative regulator *Keap1* (with a 2.85-fold increase, *p* < 0.001) in silkworms, accompanied by corresponding increases in downstream detoxification enzyme genes, indicating the activation of the CncC pathway (6F,G). Although *Keap1* was concurrently upregulated, potentially representing a feedback mechanism, the CncC pathway and its downstream targets were still activated under Pb stress. Silencing *CncC* in Pb-exposed silkworms effectively reduced *CncC* transcript levels by 75% at 48 h post-interference (Figure 7A). This knockdown significantly lowered the expression of key detoxification genes, namely *CYP18A1*, *CYP332A1*, *GSTd3*, *GSTt1*, and *UGT33D8* (Figure 7B), and led to a significant reduction in total P450 monooxygenase (Figure 7C) and GST enzyme activities (Figure 7D). These results demonstrate that *CncC* acts as a master transcriptional regulator of these detoxification enzymes during Pb stress.

To the best of our knowledge, our study provides the first functional characterization of *CncC* in mediating Pb-induced detoxification in a lepidopteran model. We demonstrate that *CncC* is essential for activating the expression of detoxification genes and their corresponding enzymatic activities, establishing it as a core defense mechanism against Pb toxicity. Collectively, these findings establish *CncC* as an indispensable master regulator orchestrating the detoxification response to Pb toxicity in silkworms, highlighting its central importance in the lepidopteran defense against heavy metal stress.

### 3.8. CncC Silencing Exacerbates Pb-Induced Apoptosis

**Figure 8:**
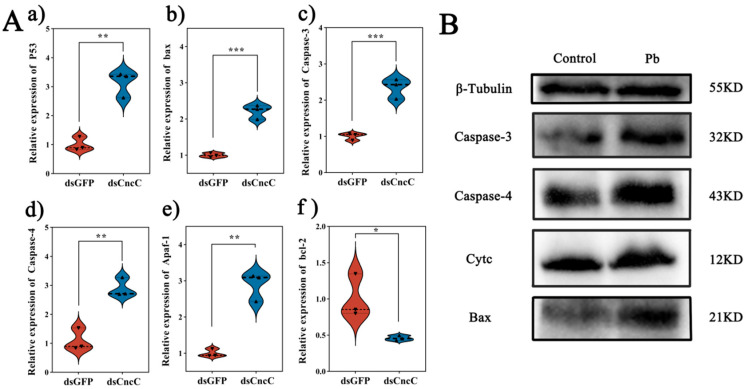
Impact of CncC knockdown on apoptosis-related genes and proteins in *B. mori* fat body. (**A**) Relative expression levels of apoptosis-related genes in dsCncC versus dsGFP groups. (**B**) Western blot analysis of apoptosis-related proteins in control and Pb-exposed groups. Data are presented as mean ± SEM (n = 3). Significant differences are indicated by asterisks (* *p* < 0.05, ** *p* < 0.01, *** *p* < 0.001; Student’s *t*-test).

Under Pb stress, silencing *CncC* led to markedly intensified apoptotic signaling in the fat body. Specifically, silencing *CncC* caused significant upregulation of pro-apoptotic genes. The expression of *p53* increased by 3.1-fold (*p* < 0.01), *Bax* by 2.2-fold (*p* < 0.001), *Caspase-3* by 2.3-fold (*p* < 0.001), *Caspase-4* by 2.9-fold (*p* < 0.01), and *Apaf-1* by 3.1-fold (*p* < 0.01), while the anti-apoptotic gene *Bcl-2* saw a 54% downregulation (*p* < 0.05) (Figure 8A). Consistently, at the protein level, the accumulation of Cytc and the levels of Bax protein and cleaved Caspase-3/4 were significantly increased (Figure 8B).

**Figure 9:**
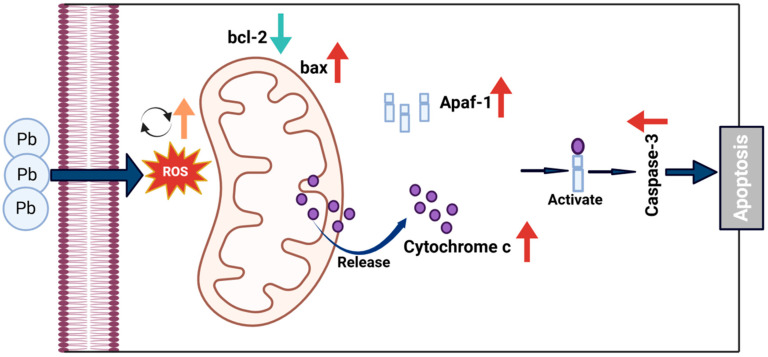
Proposed mechanism of Pb-induced apoptosis via mitochondrial pathway in B. mori. Pb exposure increases ROS generation, leading to dysregulation of Bcl-2 family proteins with decreased anti-apoptotic Bcl-2 and increased pro-apoptotic Bax. This imbalance triggers mitochondrial outer membrane permeabilization, allowing cytochrome c release from mitochondria to cytosol. In the cytosol, cytochrome c binds to Apaf-1 to form the apoptosome, activating executioner caspase-3 and leading to apoptosis.

We suggest a potential mechanism underlying these findings. *CncC* knockdown may disrupt cellular redox balance, increasing mitochondrial membrane permeability and triggering *Cytc* release. The released *Cytc* likely plays a crucial role in causing a significant rise in DNA fragmentation and maintaining *p53* activation. Consequently, escalated DNA damage and sustained p53 activation may surpass the cell’s anti-apoptotic defense mechanisms, leading to irreversible activation of the caspase cascade and apoptosis initiation (Figure 9). These results were also supported by the Western blot analysis (Figure 8B), which showed increased levels of cleaved Caspase-3 and Caspase-4 in the CncC-silenced group. Overall, these findings highlight the critical role of CncC in protecting cells from Pb-induced apoptosis by maintaining redox balance and suppressing apoptotic signaling.

## 4. Discussion

Our study demonstrates that Pb exposure induces severe histological alterations and triggers mitochondrial-mediated apoptosis in the silkworm fat body. We observed adipocyte swelling, degradation of the basal lamina, and activation of the Cytc–Caspase cascade (Figure 1 and Figure 2), indicating apoptotic cell death. This aligns with previous reports in Drosophila, where Pb induces oxidative stress-driven apoptosis through mitochondrial membrane permeabilization [47,48]. The molecular signature of apoptosis was further supported by the upregulation of pro-apoptotic genes (*Bax*, *p53*) and downregulation of the anti-apoptotic gene *Bcl-2*. The increase in Caspase-3 protein levels in Pb-exposed fat bodies is consistent with findings in *Apis mellifera* under cadmium stress [49], highlighting the conservation of mitochondrial-mediated apoptotic pathways in insects exposed to heavy metals.

Concomitantly, elevated ROS levels and increased MDA content (Figure 3) suggest that Pb disrupts redox homeostasis within the fat body. This is consistent with Pb’s role as a redox-active transition metal that interferes with mitochondrial electron transport, thereby promoting excessive ROS generation [20,49,50]. The activation of key antioxidant enzymes (SOD, CAT) reflects a compensatory defense mechanism [23,41]. However, the significant depletion of GSH suggests that detoxification, possibly via GSH–Pb complexation, imposes a metabolic burden by depleting this vital intracellular antioxidant. Elevated MDA indicates direct oxidative damage [49], while GSH depletion highlights the metabolic cost of detoxification [33]. These dual processes explain the observed histological injury and link Pb exposure to cellular dysfunction in the fat body. Our findings underscore the fat body as a primary target for Pb toxicity in insects and clarify the roles of oxidative stress and mitochondrial apoptosis in this damage.

Transcriptomic analysis identified 1,418 DEGs, which were significantly enriched in glutathione metabolism, cytochrome P450 signaling, and ABC transporters (Figure 4 and Figure 5). These findings mirror the detoxification strategies observed in other insects [27,29,31], indicating that the silkworm fat body may utilize similar mechanisms to deal with toxic substances. The increase in P450 activity and the upregulation of *CYP18A1*/*CYP332A1* suggest an enhanced Phase I metabolism (Figure 7). The induction of *GSTd3*/*GSTt1* plays a crucial role in the subsequent Phase II conjugation process. This coordinated upregulation of Phase I and Phase II enzymes is analogous to the response in Apis mellifera to pesticide exposure, highlighting the evolutionary conservation of the detoxification machinery [24,51,52].

CncC, a key transcription factor, is upregulated 7.21-fold under Pb stress (Figure 7). RNAi experiments show that CncC silencing reduces detoxification enzyme activities, confirming it as a master regulator of detoxification. This aligns with prior Drosophila studies, where CncC controlled *CYP6a8* expression under xenobiotic stress [24]. It should be noted that Keap1, a repressor of CncC, was paradoxically upregulated by 2.85-fold. This may reflect a feedback mechanism that serves to fine-tune the intensity of detoxification. A similar phenomenon has been observed in the mammalian Nrf2/Keap1 system [53]. In the mammalian Nrf2/Keap1 system, when cells are exposed to oxidative stress or toxic substances, Nrf2 is released from Keap1-mediated inhibition and translocates to the nucleus to activate the expression of antioxidant and detoxification genes [54,55]. Once the stress is alleviated, Keap1 can re-suppress Nrf2 activity to restore cellular homeostasis [18,34]. In the case of silkworms, the upregulation of *Keap1* may act in a similar way, helping to balance the detoxification response and prevent excessive activation of the detoxification system.

CncC plays a critical role in defending against Pb toxicity. Silencing CncC significantly exacerbates Pb-induced apoptosis (Figure 8), highlighting its dual function. CncC maintains cellular redox balance by transcriptionally activating key antioxidant genes, such as SOD and TRX [45,46,56]. At the same time, it suppresses pro-apoptotic signaling pathways [4]. After *CncC* knockdown, *p53* expression increases by 3.1-fold, indicating that CncC deficiency causes redox imbalance and activates DNA damage response pathways. Meanwhile, *Bax* expression rises by 2.2-fold, promoting mitochondrial outer membrane permeabilization. Cytc release increases by 1.8-fold, further showing the close link between disrupted redox regulation and the intrinsic apoptotic pathway. Cytc release also amplifies caspase cascade activation [57,58].

The fat body, which is functionally similar to the mammalian liver and integrates detoxification and energy metabolism [1,6,27], is highly sensitive to Pb. Under Pb stress, metabolic genes, such as *BmUo,* involved in purine metabolism, are downregulated (Figure 6). This metabolic reprogramming likely represents a strategic reallocation of resources to prioritize detoxification. A similar metabolic trade-off, where defense responses are favored over routine metabolism, has been observed in *B. mori* during viral infection [8]. This adaptive metabolic shift, together with CncC-mediated antioxidant and anti-apoptotic defenses, underscores the fat body’s remarkable plasticity and its integrated stress response capabilities.

This study establishes *CncC* as a central regulator of Pb detoxification in *B. mori*, significantly advancing our understanding of lepidopteran toxicology. By identifying *CYP18A1*, *GSTd3*, and *UGT33D8* as direct CncC targets, we provide potential biomarkers for environmental Pb contamination monitoring. These findings also suggest practical applications in sericulture, such as enhancing silkworm resilience through CRISPR-Cas9-mediated activation of the CncC pathway, which could help maintain productivity in Pb-affected regions. The conserved CncC/Keap1 pathway across insect orders highlights *B. mori* as a valuable ecological model for assessing Pb toxicity and risk. The correlation between CncC expression and Pb tolerance indicates its potential use in insect-based bioassays for soil contamination, offering a faster, more cost-effective, and ethical alternative to traditional vertebrate models. However, several research avenues remain open. Future studies should explore the effects of chronic, low-dose Pb exposure and tissue-specific responses. Additionally, research on interactions between Pb and other contaminants is essential for predicting combined impacts on silkworm health and ecosystems.

## 5. Conclusions

This study elucidates the molecular mechanisms of Pb-induced toxicity in silkworm fat bodies. Pb exposure induces mitochondrial pathway-mediated apoptosis and oxidative stress, upregulates antioxidant enzymes, and enhances P450 and GST activities. Transcriptomics analysis reveals numerous DEGs enriched in immune, antioxidant, and detoxification pathways. Our results highlight the transcription factor *CncC* as a central regulator in the Pb detoxification response. Pb stress increases the expression of *CncC* and its downstream genes. Silencing *CncC* reduces their expression levels and enzyme activities, and exacerbates apoptosis and redox imbalance. Overall, the CncC pathway plays a vital role in mitigating Pb toxicity, providing valuable insights for enhancing silkworm resistance to heavy metals and assessing Pb contamination risks.

## Data Availability

The raw transcriptome data have been uploaded to the NCBI database, and the accession number is PRJNA1112580.

## Supplementary Materials

The following supporting information can be downloaded at: https://www.mdpi.com/article/10.3390/insects16070699/s1, Figure S1: Gene ontology (GO) annotation of the B. mori transcriptome. The x-axis shows GO functions, the right y-axis indicates the number of genes with GO functions, and the left y-axis represents the percentage; Table S1: The primers of qRT-PCR; Table S2: Quality of clean reads; Table S3: Statistical table of sequencing data and assembly results; Table S4: Statistics of success rate of annotation; Table S5: Table of GO enrichment of all genes and DEGs; Table S6: Classification table of the unigenes annotated in COG.
