# Supplementary material for: Insights into Lead Toxicity and Detoxification Mechanisms in the Silkworm, Bombyx mori
Source: Insects. 2025 Jul 7;16(7):699. doi: 10.3390/insects16070699 (PMC12295734; doi:10.3390/insects16070699)
Supplement: Supplementary file 1 [file insects-16-00699-s001.zip › insects-3658834-supplementary.pdf]

Table S1 The primers of qRT-PCR

Primer name	Forward Primer (5'-3')	Reverse Primer(5'-3')
Actin3	CGGCTACTCGTTCACTACC	CCGTCGGGAAGTTCGTAAG
CYP18a1	TCCTTCAATTGGCCAGAAAC	ACCGTCGTTGATTTTTCCAG
CYP332A1	CCGTTCTTCTTGTTCGGAAG	GACGCGCTATTTCTGGAGTC
GSTe1	AAACAAATACGGCAAAGG	TCCACGGTGAATCAGAAA
UGT33D8	ACGTTGGAGGAATCCATCAG	GTCCCATTTCCAGAGCACAT
UGT40A1	AACGCCTGCTCCACTAAAGA	CAACAATCACGGCATCAAAC
CYP314A1	GAGAATTCAAACCGGAACGA	CCACAATCCTTTGGCTGATT
UGT2	GCTTCTGTTTGGGTGTCGAT	AAACGGCGTTATGTGTGTCA
UGT33D4	TGTGACCACTGACCCTGTGT	TCCTTAGCAACGCTTGGACT
Caspase-3	GAAATACGCTACGACATAC	CGACTTCAAAGCCAAAC
Caspase-4	CAGGAGAACATCGGAATA	TTCTTAGATGACTCGCTTAT
Apaf-1	GATACGGACGTTGTGGC	CTTTCGCATACGATGGA
bcl-2	TGCCCTTTCAACAGACT	ATGCCACAACATTACCG
Bax	GCTCTGCTCGGCACTAC	CCCATTCTGCGTTTCTC
P53	GACTTCGCTGTCCAGTTCAA	CCAGCACGGAGTACCAGTAG
CncC	TCCCGTCAAGTACGAGTGTC	GTGAGTCGGCGAACCCT
Daxx	CGTTCCCTGATTTCCAT	GACCTGCACAGCATTCC
CAT	AATGTCGGCGGAGATGTAGACC	GCAGCAGCATCCTTGAGGTGAT
GST03	CACTCCCACGACCCTTAC	CATGTAGTCCAGCATTCCAG
FOXO	CACCAATACCAGCGAAGAA	CAGTGTGAGCCTGTTGTC
ATG8	AAGGCTAGGCTTGGAGAC	CAGATGTGGGTGGAATGA
Keap1	ATGACCTGCCTCCGATTAGT	TCCAACTTCCAACACGACATC
AMY	ATCCTGCTGTTCCTTACGGC	TGTCAGGGCAGCAGTTGTAG
dsCncC	GCGTAATACGACTCACTATAGGG GAACAAAGTGGCAGCACAGA	GCGTAATACGACTCACTATAGGGCG TAGTGCTTGGCTTTCCTC
dsGFP	GCGTAATACGACTCACTATAGGG AGAAGAACTTTTCACTGG	GCGTAATACGACTCACTATAGGGTG AACGGATCCATCTTC

Table S2 Quality of clean reads.

Samples	Clean Reads	Clean Bases	GC(%)	Q30(%)
CK	21816830	6532954743	47.46%	95.37%
Pb	21267028	6367523461	47.75%	95.60%

Table S3 Statistical table of sequencing data and assembly results.

Sample	Total Reads	Mapped Reads	Uniq Mapped Reads	Multiple Map Reads
CK	43633660	40268048(92.29%)	38594919(88.45%)	1673129(3.83%)
Pb	42534056	40237638(94.60%)	38161831(89.72%)	2075807(4.88%)

Table S4 Statistics of success rate of annotation.

Type	Total	COG	GO	KEGG	KOG	NR	Pfam	Swiss-Prot	eggNOG
unigenes	15842	3388	10692	10811	8260	15725	10524	6825	10874
DEGs	1391	401	1053	1052	880	1384	1112	724	1088

Table S5 Table of GO enrichment of all genes and DEGs.

GO·ID	Term	Unigene	DEGs
GO:0005622	intracellular	2795	249
GO:0032991	protein-containing complex	1166	86
GO:0110165	cellular anatomical entity	5852	547
GO:0003824	catalytic activity	3688	378
GO:0005198	structural molecule activity	374	21
GO:0005215	transporter activity	692	92
GO:0005488	binding	4912	482
GO:0016209	antioxidant activity	44	4
GO:0038024	Cargo receptor activity	8	1
GO:0045182	translation regulator activity	58	2
GO:0060089	molecular transducer activity	254	16
GO:0098772	molecular function regulator	305	31
GO:0140104	molecular carrier activity	3	0
GO:0140110	transcription regulator activity	286	35
GO:0140299	small molecule sensor activity	2	0
GO:0000003	reproduction	242	25
GO:0002376	immune system process	81	8
GO:0007610	behavior	97	9
GO:0008152	metabolic process	3789	387
GO:0009987	cellular process	5157	493
GO:0022414	reproductive process	230	24
GO:0022610	biological adhesion	104	3
GO:0023052	signaling	768	71
GO:0032501	multicellular organismal process	772	62
GO:0032502	developmental process	621	59
GO:0040007	growth	43	5
GO:0040011	locomotion	173	18
GO:0043473	pigmentation	21	3
GO:0044419	interspecies interaction between organisms	74	11

GO:0048511	rhythmic process	25	5
GO:0050896	response to stimulus	1194	104
GO:0051179	localization	1375	149
GO:0051703	intraspecies interaction between organisms	2	0
GO:0051704	multi-organism process	234	24
GO:0065007	biological regulation	2164	198
GO:0098754	detoxification	45	4

Table S6 Classification table of the unigenes annotated in COG.

#ID	Class_Name	Numbers	Percentage(%)
J	Translation, ribosomal structure and biogenesis	339	10.01%
A	RNA processing and modification	5	0.15%
K	Transcription	53	1.56%
L	Replication, recombination and repair	179	5.28%
B	Chromatin structure and dynamics	7	0.21%
D	Cell cycle control, cell division, chromosome partitioning	40	1.18%
Y	Nuclear structure	0	0.00%
V	Defense mechanisms	140	4.13%
T	Signal transduction mechanisms	239	7.05%
M	Cell wall/membrane/envelope biogenesis	131	3.87%
N	Cell motility	13	0.38%
Z	Cytoskeleton	15	0.44%
W	Extracellular structures	6	0.18%
U	Intracellular trafficking, secretion, and vesicular transport	18	0.53%
O	Posttranslational modification, protein turnover, chaperones	402	11.87%
C	Energy production and conversion	169	4.99%
G	Carbohydrate transport and metabolism	379	11.19%
E	Amino acid transport and metabolism	232	6.85%
F	Nucleotide transport and metabolism	107	3.16%
H	Coenzyme transport and metabolism	91	2.69%
I	Lipid transport and metabolism	399	11.78%
P	Inorganic ion transport and metabolism	158	4.66%
Q	Secondary metabolites biosynthesis, transport and catabolism	291	8.59%
R	General function prediction only	435	12.84%
S	Function unknown	58	1.71%
