# Supplementary figures and images for: Insights into Lead Toxicity and Detoxification Mechanisms in the Silkworm, Bombyx mori
Source: Insects. 2025 Jul 7;16(7):699. doi: 10.3390/insects16070699 (PMC12295734; doi:10.3390/insects16070699)

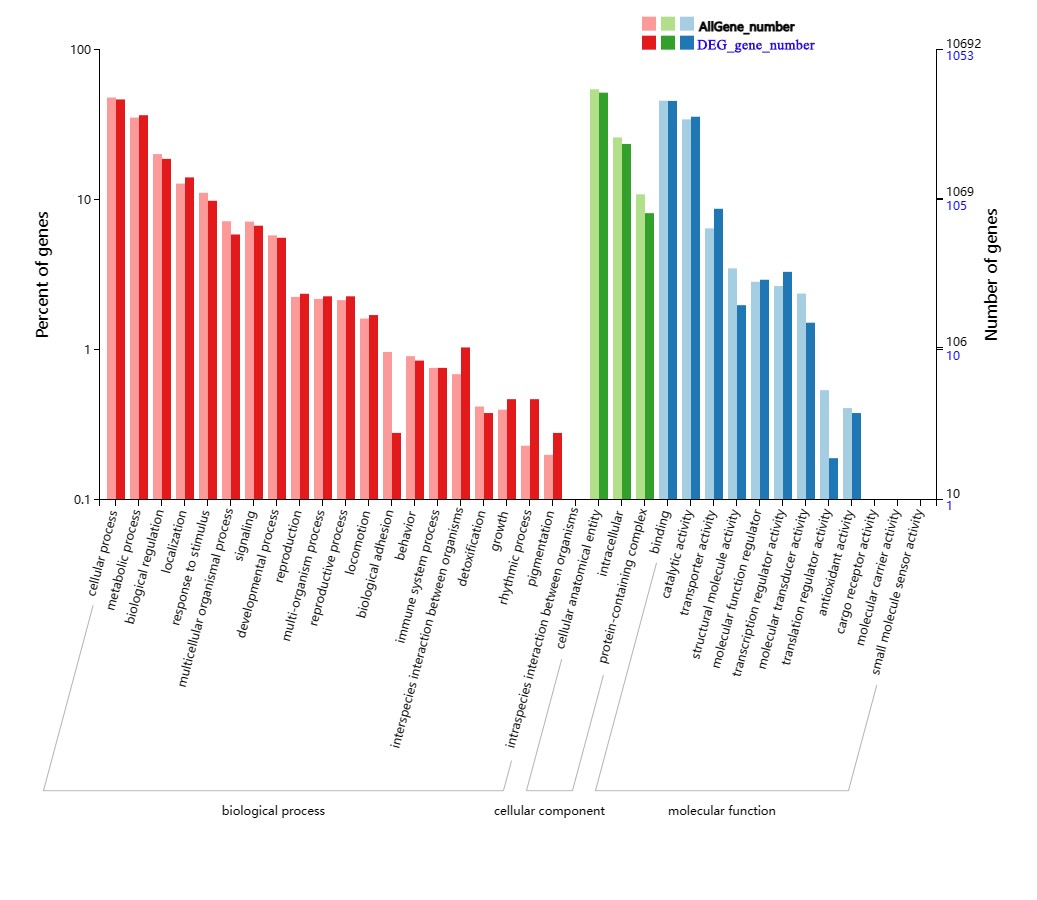

Supplement: Supplementary file 1 [file insects-16-00699-s001.zip › Figure S1.jpg]
